# The OsSec18 complex interacts with P0(P1-P2)_2_ to regulate vacuolar morphology in rice endosperm cell

**DOI:** 10.1186/s12870-014-0324-1

**Published:** 2015-02-17

**Authors:** Yunfang Sun, Tingting Ning, Zhenwei Liu, Jianlei Pang, Daiming Jiang, Zhibin Guo, Gaoyuan Song, Daichang Yang

**Affiliations:** State Key Laboratory of Hybrid Rice and College of Life Sciences, Wuhan University, Luojia Hill, Wuhan, Hubei Province 430072 China

**Keywords:** OsSec18, Os60sP0(P1-P2)_2_ complex, Vacuole fusion, Rice endosperm

## Abstract

**Background:**

Sec18p/N-ethylmaleimide-sensitive factor (NSF) is a conserved eukaryotic ATPase, which primarily functions in vesicle membrane fusion from yeast to human. However, the function of the *OsSec18* gene, a homologue of NSF in rice, remains unknown.

**Results:**

In the present study, we investigated the function of *OsSec18* in rice and found that *OsSec18* complements the temperature-sensitive phenotype and interferes with vacuolar morphogenesis in yeast. Overexpression of *OsSec18* in rice decreased the plant height and 1000-grain weight and altered the morphology of the protein bodies. Further examination revealed that OsSec18 presented as a 290-kDa complex in rice endosperm cells. Moreover, Os60sP0 was identified a component of this complex, demonstrating that the OsSec18 complex contains another complex of P0(P1-P2)_2_ in rice endosperm cells. Furthermore, we determined that the N-terminus of OsSec18 can interact with the N- and C-termini of Os60sP0, whereas the C-terminus of OsSec18 can only interact with the C-terminus of Os60sP0.

**Conclusion:**

Our results revealed that the OsSec18 regulates vacuolar morphology in both yeast and rice endosperm cell and the OsSec18 interacts with P0(P1-P2)_2_ complex in rice endosperm cell.

**Electronic supplementary material:**

The online version of this article (doi:10.1186/s12870-014-0324-1) contains supplementary material, which is available to authorized users.

## Background

Sec18p/N-ethylmaleimide-sensitive factor (NSF) is a conserved ATPase required for vesicle membrane fusion in eukaryotes. In yeast and mammalian cells, the mechanism of vesicle membrane fusion, which is mediated by Sec18p/NSF and the soluble NSF attachment protein (SNAP) receptor (SNARE) complex, has been extensively investigated. NSF assembles with SNAP and SNAREs to form a 20S SNARE fusion complex that mediates membrane fusion between vesicles [1]. This 20S fusion complex is disassembled by NSF via ATP hydrolysis [2]. During this process, Sec18p/NSF, acting as a SNARE chaperone, binds to SNARE complexes, disassembling them and facilitating SNARE recycling by utilizing the energy from ATP hydrolysis. The rate of Sec18p/NSF-mediated disassembly correlates to the SNARE-activated ATPase activity of NSF [3].

NSF is also involved in protein trafficking [4-7]. Previous studies have indicated that NSF binds directly to the C-terminal tail of the GluR2 subunit of the alpha-amino-3-hydroxy-5-methyl-4-isoxazolepropionate (AMPA) receptor in a SNAP-dependent manner to regulate the function of these receptors [6,7]. McDonald et al. have found that NSF can bind to β-arrestin1 and plays a hitherto role in facilitating clathrin coat-mediated internalization of G protein-coupled receptors [8]. Cong *et al*. have confirmed that NSF can bind to β2 adrenergic receptors (β2-ARs) at the final three amino acids in the C-terminal tail of these receptors, thereby regulating receptor recycling [4].

To date, very limited information about Sec18p/NSF and SNARE complexes in plants is available. Sato *et al*. have cloned a homolog of NSF from tobacco, designated as *NtNSF-1*, which encodes a 739-aa protein that displays ATP binding capacity [9]. Hugueney *et al*. have investigated a plastid fusion and/or translocation factor (Pftf) in *Capsicum annuum* and demonstrated that it functions in vesicle fusion in an ATP-dependent manner. However, *Pftf*, which encodes a 72-kDa protein, was only expressed in leaves and young fruit in red peppers [10]. Bioinformatic analysis indicated that its cDNA sequence displays 53% and 51% homology with yeast Sec18p and mammalian NSF, respectively. However, the functions of *OsSec18,* a homolog of Sec18p/NSF in rice, remain unknown.

More recently, some studies have indicated that the proteins involved in protein sorting play important roles in plant development. Vacuolar protein sorting 29 (VPS29) is a component of a retromer complex that recycles the vacuolar sorting receptor VPS10 from the pre-vacuolar compartment (PVC) to the Golgi complex. In *Arabidopsis*, the VPS29 homolog Maigo1 (MAG1)/AtVPS29 is ubiquitously expressed in various organs, including leaves, roots, flowers and developing seeds [11]. The *MAG1* mutant (*mag1*) exhibits a dwarf phenotype, suggesting that it may play a significant role in plant growth and development [12]. Furthermore, VPS29 is involved in endosome homeostasis, PIN protein cycling, and VSR recycling from the PVC to the trans-Golgi network (TGN) during the trafficking of soluble proteins to the lytic vacuole (LV) [13,14]. Moreover, the protein sorting protein 45 (VPS45p), a member of the Sec1p family, is involved in vesicle-mediated protein trafficking in various organelles of the endomembrane system [15,16]. Bassham *et al*. have found that AtVPS45p co-localized with an epidermal growth factor receptor-like protein (AtELP) in *Arabidopsis* in the TGN and that AtVPS45p functions in the transport of proteins to the vacuole in plants [15,16]. However, the relevance of *OsSec18* and PVC remains to be determined in rice.

Ribosomal acid protein P0 as a component of P0 (P1-P2)_2_ complex, functioning on protein synthesis as a subunit of 60s ribosomes [17,18]. The C-terminus (199-258aa) of P0 binds to the (P1-P2) small complex [19], while the N-terminus (44-67aa) of P0 interacts to the RNA molecule after P0(P1-P2)_2_ complex formed [20]. Mutation of P0 gene affects the ribosome activity and viability of *Saccharomyces cerevisiae* [21]. Barnard *et al*. and Kondoh *et al*. have found that the human ribosomal phosphoprotein P0 may be implicated in human colorectal cancer progression [22,23]. Recently, Chang *et al*. have found that overexpression of P0 protein might cause oncogenesis in breast and liver tissues by partially inhibiting GCIP-mediated tumor suppression [19]. All these results suggest that P0 protein is important for the protein synthesis as well as other cellular functions, such as oncogenesis [17-19]. Rice Os60sP0 is 60% homologous to human 60sP0 in DNA sequences and 53% homologous in amino acids sequences. When compared with yeast, the homology is 54% and 46%, respectively [24]. However, the functions of P0 (P1-P2)_2_ complex in rice have not been previously reported.

In the present study, we investigated the function of *OsSec18* in rice and found that it can complement the temperature-sensitive phenotype but cannot restore vacuolar morphology in yeast. This result suggests that the *OsSec18* gene may perform other unknown functions than in yeast. Overexpression of the *OsSec18* gene in rice decreased the plant height and 1000-grain weight, and changed the morphology of the protein bodies. Further studies demonstrated that OsSec18 is a component of a 290-kDa complex in rice endosperm cells. Moreover, Os60sP0 was identified as a component of this complex, revealing that the OsSec18 complex contains another complex of P0(P1-P2)_2_ in rice endosperm cells. Furthermore, we determined that the N-terminus of OsSec18 interacts with the N- and C-termini of Os60sP0, whereas the C-terminus of OsSec18 interacts only with the C-terminus of Os60sP0. We proposed a molecular model for the interaction between OsSec18 and Os60sP0.

## Results

### The expression profile of *OsSEC18* in rice

Although Sec18 has been extensively studied in yeast and mammals, its functions in plants remain unknown. To investigate the function of *Sec18* in rice, we first searched the rice genome database (www.gramene.org). An *OsSec18* gene (GenBank No. Os05g0519400) is homologous to *SEC18* in yeast. OsSec18 shares 46%, 45%, 75% and 37% homology with tobacco NSF, yeast Sec18p, human NtNSF-1 and *Capsicum annuum* Pftf, respectively (Additional file 1: Figure S1 and Additional file 2: Figure S2). OsSec18 contains two AAA ATP domains at the C terminus and the middle region of the amino acids sequence, and it displays ATP-binding and nucleotide-binding nucleoside-triphosphatase activity.

**Figure 1:**
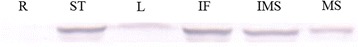
**Tissue-specific expression patterns of the OsSec18 protein.** R, root; ST, stem; L, leaf; IF, inflorescence; IMS, immature seed; MS, mature seed.

To explore the expression profile of OsSec18 in rice, we analyzed various tissues and organs via Western blot analysis. The results revealed that OsSec18 expressed in leaf, stem, inflorescence, and immature and mature seeds but not in root. The highest expression level was found in stem, inflorescence and immature seed (Figure 1).

Interestingly, we found three isoforms or modifications of OsSec18. OsSec18 displayed the lowest molecular mass in inflorescence and immature seed, followed by mature seed and stem, and the highest mass in leaf. These results indicated that OsSec18 is expressed as distinct isoforms or is modified in a tissue-specific manner, implying that these isoforms or modifications may play distinct roles in different organs or tissues.

### *OsSec18* does not completely complement the function of vesicle fusion in the yeast *sec18* mutant

To investigate whether *OsSec18* performs the same functions in vesicle fusion as in yeast, a genetic complementation assay was conducted. The *OsSec18* gene driven by the *CaMV35S* promoter was introduced into the yeast temperature-sensitive Sec18p mutant strain sey5186 (*MAT sec18-1 ura3-52 leu2-3, 112 GAL*^*+*^) and the wild-type strain sey6210 (*MAT ura3-52 leu2-3, 112 his3-200 trp1-901 lys2-801 suc2-9*). sey5186 overexpressing *OsSec18* grew well at 37°C, whereas the mutant sey5186 alone did not grow (Table 1).

**Table 1:** **Yeast complementation assays**

**Selective medium**	**Growth temperature**		
	**23°C**	**37°C**	
	**sey5186**	**sey5186**	**sey6210**
ura+	+	-	+
ura-Gal-	+	-	+
ura-Gal+	+	+	+

Note: sey5186 is a temperature-sensitive *sec18* gene mutant strain that grows slowly at 23°C but does not survive at 37°C; sey6210, a wild-type strain, grows normally at 37°C.

**Figure 2:**
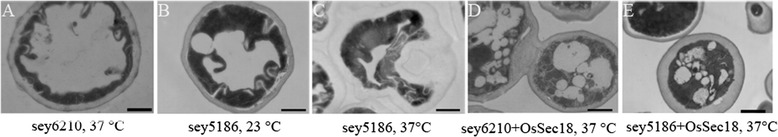
**EM analysis of sey5186 and sey6210. A**, Wild-type sey6210 at 37°C; **B**, Sec18 mutant sey5186 at 23°C; **C**, Sec18 mutant sey5186 at 37°C after 2 hours; **D**, sey6210 transfected with *OsSec18* at 37°C after 2 hours; **E**, sey5186 transfected with *OsSec18* at 37°C after 2 hours.

These results showed that the *OsSec18* gene complemented the function of the yeast temperature-sensitivity of the yeast Sec18p mutant. Furthermore, we examined the morphologies of the vacuoles in sey5186 overexpressing *OsSec18*. No clear differences in vacuole morphology were found between sey5186 grown at 23°C and the wild-type strain sey6210 grown at 37°C (Figure 2A, B), but the shapes of vacuoles appeared to be sunken in sey5186 grown at 37°C (Figure 2C). However, an significant difference in vacuolar morphology were observed between sey5186 grown at 23°C and sey5186 overexpressing *OsSec18* grown at 37°C (Figure 2B, and E). The vacuoles in sey5186 overexpressing OsSec18 were smaller compared with those in sey6210 grown at 37°C as sey5286 grown at 23°C (Figure 2A, B, and E). Moreover, the same vacuolar morphologies were detected in sey6210 overexpressing *OsSec18* grown at 37°C and in sey5186 overexpressing *OsSec18* (Figure 2D and E).

Clearly, vesicle fusion was disturbed when *OsSec18* was expressed in yeast cells. These results showed that the *OsSec18* gene not only restored the ability of sey5186 to grow at 37°C but also interfered with vesicle fusion, thus altering vacuolar morphology in yeast. This result suggests that *OsSec18* performs nearly the same growth-related function as Sec18/NSF in yeast, but *OsSec18* also interrupts vesicle fusion and vacuolar morphology.

### Overexpression of *OsSec18* alters the morphology of the protein bodies

To explore the function of *OsSec18* in rice, we constructed an overexpression vector driven by the *CaMV35S* promoter and transformed this vector into the rice genome via biolistic bombardment. Nine independent transformants were obtained. The *OsSec18-*positive line 124-5-7 was identified via Western blotting and PCR, and then used for further experiments. The phenotypic analyses revealed that the plant height significantly decreased by 17.12% and the 1000-seed weight decreased by 19.62% in the *OsSec18*-overexpressing line (Table 2), suggesting that *OsSec18* is involved in rice spikelet development.

**Table 2:** **Phenotypic analyses of transgenic and wild-type rice**

**Variety/line**	**Phenotype**	**Plant height (cm)**	**+/− (%)**	**1000-grain weight (g)**	**+/− (%)**
TP	Control	101.45		20.9	
124-5-7	Overexpressing	84.08**	−17.12	18.1**	−13.40
124-8-37	Overexpressing	91.77**	−9.54	16.8**	−19.62

**P < 0.01.

**Figure 3:**
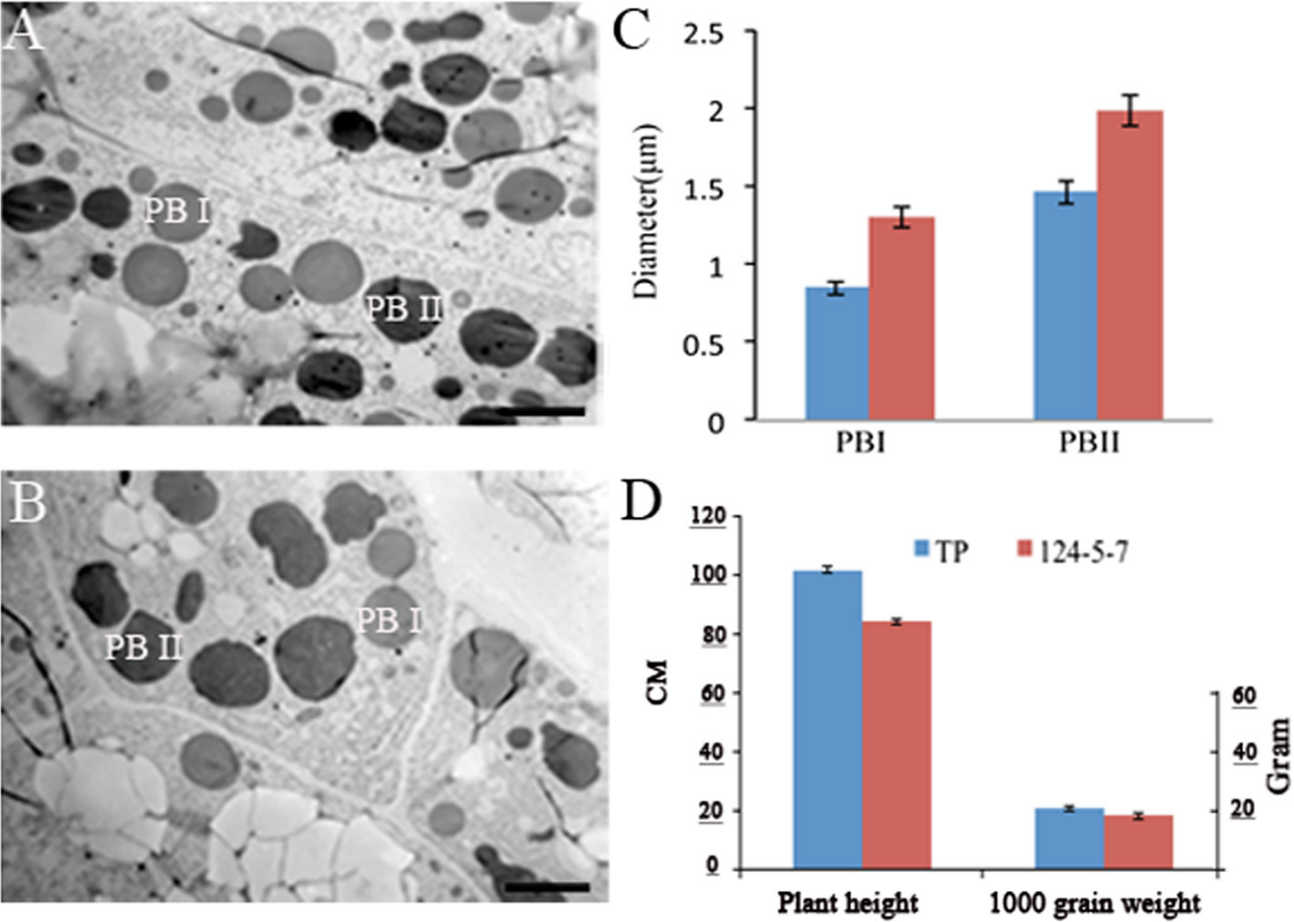
**Phenotypic comparison of the grains and EM analysis of the endosperms between wild-type and transgenic plants.** (**A** and **B**) EM analysis of the endosperm. **A**, The wild-type line; **B**, The *OsSec18* overexpressing transgenic line 124-5-7; **C**, Sizes of PB I and PB II in wild-type and transgenic plants, which generated from 25 protein bodies; **D**, Plant height and 1000-grain weight analyses of the wild-type and transgenic plants.

Furthermore, based on the finding of a change in vacuolar morphology in yeast overexpressing *OsSec18*, we explored whether the morphology of the protein bodies was affected. We examined the subcellular morphology of the protein bodies in endosperm cells. The protein body II (PBII) and protein body I (PBI) sizes in line 124-5-7 were larger than those of the wild-type line. The size of PBI in the *OsSec18*-overexpressing line was increased by 30.17%, and that of PBII was increased by 25.75% (Figure 3A and B). There was a positive correlation between the agronomic phenotypes and the sizes of the protein bodies (Table 2 and Figure 3). These results again showed that *OsSec18* is involved in protein storage vacuolar (PSV) morphology in rice endosperm cells.

### OsSec18 is a component of a 290-kDa complex in rice endosperm cells

**Figure 4:**
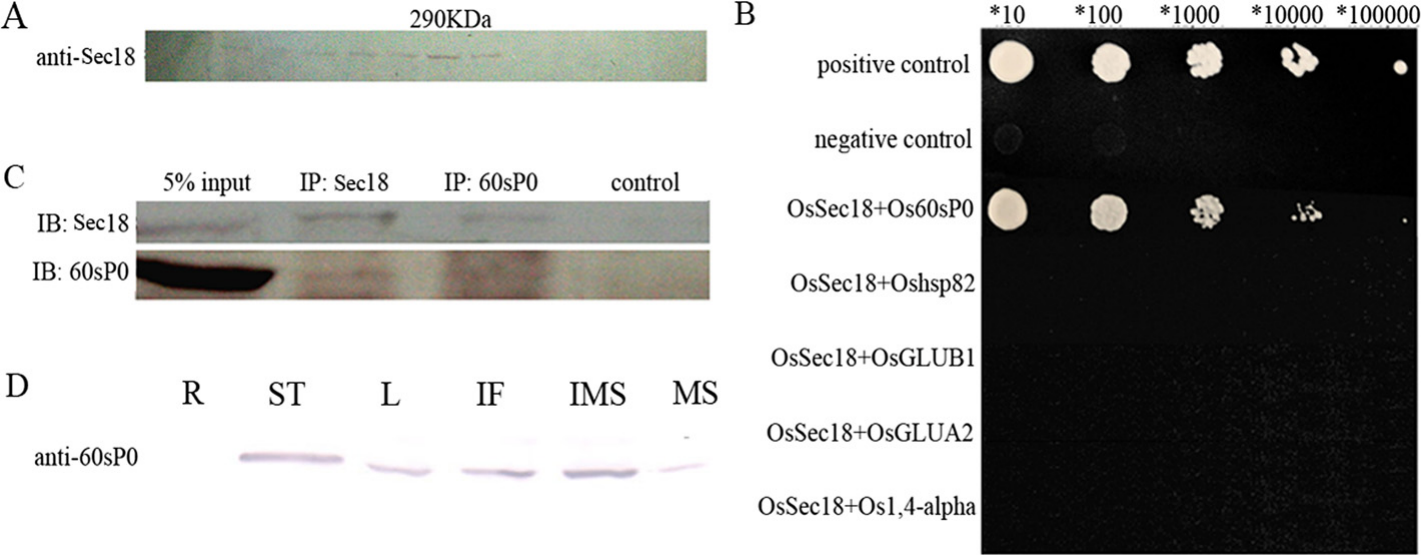
**The OsSec18 protein interacts with the Os60sP0 protein both** ***in vitro*** **and** ***in vivo.*** **A**, The OsSec18 protein complex in rice endosperm. Crude protein extract from rice immature endosperm was loaded on a Sepharose 300 gel filtration column and detected via Western blotting using anti-Sec18 serum; **B**, Yeast two-hybrid analysis of OsSec18 and Os60sP0p. Positive, co-transformation with the positive plasmids pGBKT7-53p and GADT7-RecT; negative, co-transformation with the negative plasmids pGBKT7-Lam and GADT7-RecT; **C**, The Co-IP results using serum against OsSec18 or Os60sP0. The negative control is the antibody against OsSec18 or Os60sP0p in RIPA buffer in the absence of the crude protein extract; **D**, The tissue-specific expression patterns of the OsSec18 protein. R, root; ST, stem; L, leaf; IF, inflorescence; IMS, immature seed; MS, mature seed.

To further investigate the functions of *OsSec18* in PSV morphology during endosperm development, we hypothesized that OsSec18 might contribute to protein trafficking or docking in a complex form in rice endosperm cell. To test this hypothesis, we performed size exclusion chromatography (SEC) and co-immunoprecipitation (Co-IP). As shown in Figure 4A, a 290-kDa protein complex was identified via SEC using the serum against OsSec18. To identify the components of this protein complex, the fraction corresponding to this 290-kDa complex was separated via sodium dodecyl sulfate-polyacrylamide gel electrophoresis (SDS-PAGE). The proteins were recovered and sequenced via MALDI-TOF mass spectrometry. Five proteins, heat shock protein 81–1 (hsp82), glutelin type B1 (GLUB1), glutelin type A2 (GLUA2), 60S acidic ribosomal protein P0 (Os60sP0p) and 1,4-alpha-glucan branching enzyme were identified. To confirm the participation of these proteins in this complex, we performed a yeast two-hybrid assay. The results indicated that only Os60sP0p interacted with OsSec18, and no interaction was detected between OsSec18 and the other four proteins (Figure 4B). To verify the results of the yeast two-hybrid assay, we performed Co-IP. As shown in Figure 4C, OsSec18 was detected in the output precipitated using the Os60sP0p antibody, and conversely, Os60sP0p was detected in the output precipitated using the OsSec18 antibody. Furthermore, we examined the expression patterns of Os60sP0p in various tissues, and we found the same expression patterns as those of OsSec18 (Figure 4D). Taken together, our results demonstrate that Os60sP0p is a component of the OsSec18 complex in rice endosperm cells.

**Figure 5:**
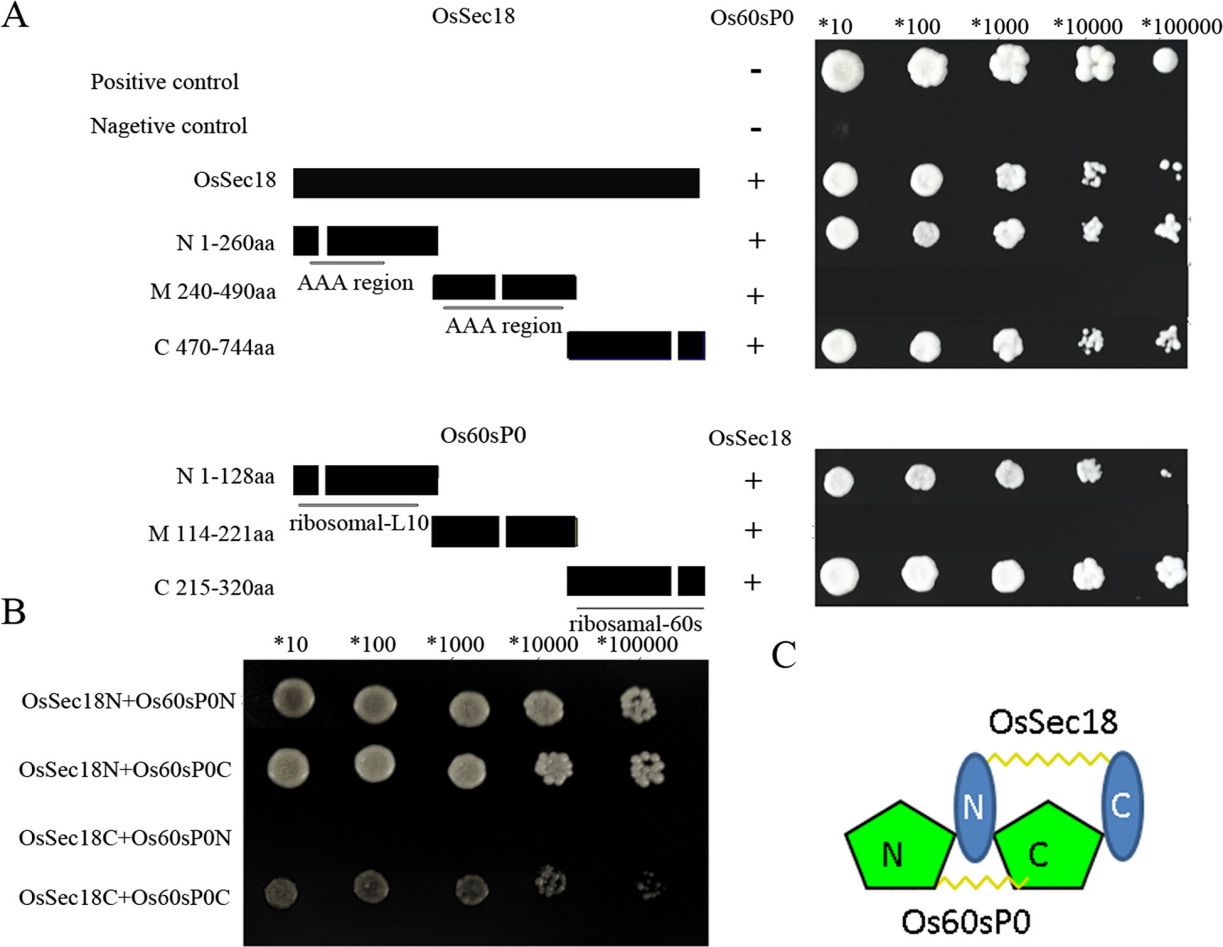
**The pattern of interactions between the OsSec18 and Os60sP0 proteins.** (**A** and **B**) Yeast two-hybrid analysis of various OsSec18 and Os60sP0p constructs. Positive, co-transformation with the positive plasmids pGBKT7-53p and GADT7-RecT; negative, co-transformation with the negative plasmids pGBKT7-Lam and GADT7-RecT. **C**, An interaction model for the OsSec18 and Os60sP0 proteins.

To further characterize which domains of OsSec18 interact with the domains of Os60sP0p, we constructed a series of vectors containing different truncated fragments of both OsSec18 and Os60sP0p by inserting random deletion mutations. Reciprocal hybrids of these truncated fragments were generated via yeast two-hybrid assays (Figure 5A). We found that the N-terminus (1–260 aa) and C-terminus (470–744 aa) of OsSec18 interacted with the full-length Os60sP0p, and the N-terminus (1–128 aa) and C-terminus (215–320 aa) of Os60sP0p interacted with the full-length OsSec18 (Figure 5A and B). The middle fragments did not interact with each other. Further examination revealed that both the N- and C-termini of OsSec18 interacted with the N-terminus, but not the C-terminus, of Os60sP0p. Moreover, the C-terminus of OsSec18 only interacted with the C-terminus of Os60sP0p (Figure 5B). These results indicated that the N-terminus head and the C-terminus tail of OsSec18 bind to the N-terminus head of Os60sP0p, whereas the C-terminus tail of Os60sP0p only binds to the C-terminus tail of OsSec18 (Figure 5C). These results confirmed that OsSec18 and Os60sP0p are constituents of the same protein complex in endosperm cells, indicating that the N- and C-termini of OsSec18 can recruit the N-terminus of Os60sP0p and that conversely, the C-terminus of Os60sP0p can recruit the C-terminus of OsSec18.

### P0(P1-P2)_2_ is a component of the OsSec18 complex *in vivo*

**Figure 6:**
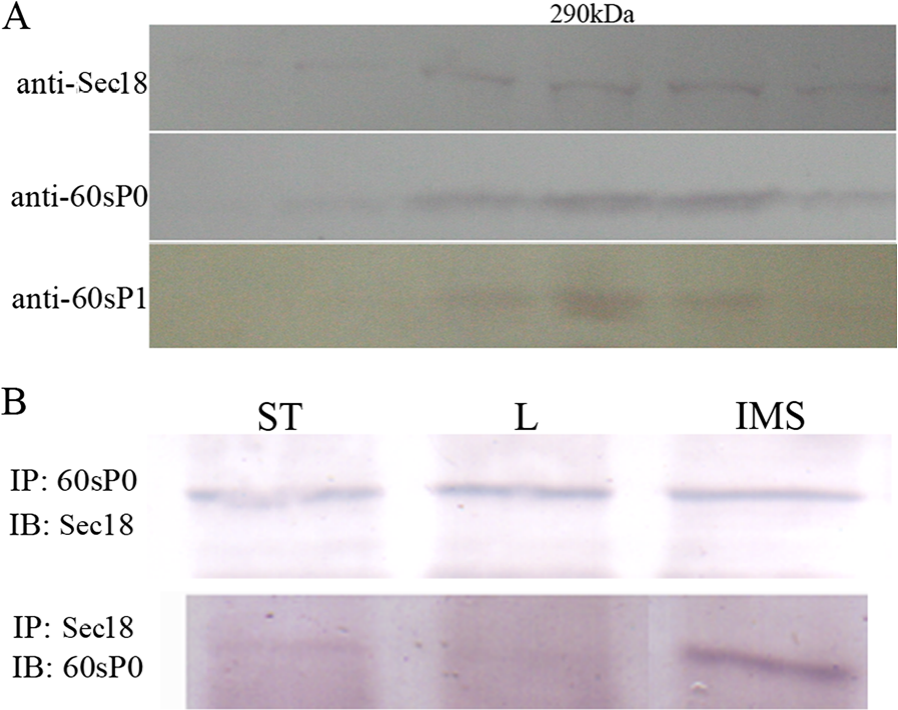
**P0(P1-P2)**_**2**_ **is a component of the OsSec18 complex** ***in vivo.*** **A**, The P0(P1-P2)_2_ complex and OsSec18 are present in the same complex based on a gel-filtration experiment. The crude protein extract from rice immature endosperm was loaded on a Sepharose 300 gel filtration column and detected via Western blot using anti-Sec18 or anti-60sP0 serum; **B**, Co-IP of Os60sP0p and OsSec18 in stem, leaf and immature seed.

Previous studies showed that 60sP0p in eukaryotes can constitute heterologous complex P0(P1-P2)_2_ consisting of two P proteins, P1 and P2 [25]. The C-terminus (199–258 aa) of P0 binds to the (P1-P2) small complex [19]. The lysine-rich N-terminus (44–67 aa) can bind to RNA when the P0(P1-P2)_2_ complex is formed [20]. Our results revealed that the C-terminus of Os60sP0p binds to both the N- and C-termini of OsSec18 (Figure 5B). To explore whether heterologous P0(P1-P2)_2_ complex co-exists in the OsSec18 complex, we performed Western blot using antiserum for P1 in the eluent fractions collected during SEC. P0 and P1 were detected in the output fraction precipitated by the OsSec18 antibody, and P1 peaked at 290 kDa with OsSec18, indicating that P0(P1-P2)_2_ co-exists in the OsSec18 complex (Figure 6A). To further explore whether OsSec18 and Os60sP0 are expressed in the same complex of various tissues, we examined this complex in crude protein extracts from rice stem, leaf and endosperm via Co-IP. These results indicated that the OsSec18-Os60sP0(P1-P2)_2_ complex presents in the stem and endosperm but not in the leaf (Figure 6B), consistent with the expression pattern of OsSec18. Taken together, our results demonstrate that the heterologous complex P0(P1-P2)_2_ is a component of the OsSec18 complex.

## Discussion

Although Sec18p has been extensively studied in yeast and mammalian cells, its functions in plant vacuolar compartments remain to be determined. In this study, we found that *OsSec18* rescued a yeast temperature-sensitive mutant phenotype and affected vacuole morphology by interfering with vesicle fusion when overexpressed in either mutant sey52186 or wild-type sey6120 cells in yeast. Three isoforms of OsSec18 were found in different tissues of rice. Furthermore, our data further indicated that OsSec18 affects the morphology of PSV in rice endosperm cells. Moreover, we identified a 290-kDa complex of OsSec18 in rice endosperm and demonstrated that heterologous complex P0(P1-P2)_2_ is another component of OsSec18 complex. Our data indicate that OsSec18, along with heterologous complex P0(P1-P2)_2_, is involved in rice vacuolar morphogenesis.

Recently, Jaillais *et al.* studied vacuolar protein sorting 29 (VPS29), a single-copy gene in *Arabidopsis* that is an ortholog of *VPS29* in yeast and mammals [13]. They found that not only is the AtVPS29 protein a member of the retromer complex but also is required for endosome homeostasis, PIN protein cycling and dynamic PIN1 repolarization during development. Although the interaction among OsSec18, Os60sP0 and PVC had not been reported previously, several studies indicated that the novel function of VPS genes rather than vacuolar fusion and protein trafficking [13,16] . In our study, we found that Os60sP0 play a novel function of vacuolar morphology than protein synthesis. In general, ribosomal acid protein P0, a component of the P0(P1-P2)2 complex, is a subunit of the 60s ribosomal complex that mediates protein synthesis [17,18]. Previous studies indicated that mutation of the *P0* gene affects ribosome activity and cell viability in *Saccharomyces cerevisiae* [21]. Barnard *et al.* have found that human ribosomal protein P0 phosphorylation is involved in the progression and biological aggressiveness of human colorectal cancer [22]. Furthermore, Kondoh *et al.* found that P0 may exert a causal effect on hepatocellular carcinoma (HCC) progression via the translational machinery due to its interaction with eukaryotic elongation factors [23]. Recently, Chang *et al.* reported that the overexpression of the P0 protein may cause tumorigenesis in breast and liver tissues, which at least partially inhibited GCIP-mediated tumor suppression [19]. Based on our data, we found that OsSec18 interacted with P0(P1-P2)2 to form an OsSec18-P0(P1-P2)2 complex. Serial deletion mutation demonstrated that OsSec18 binds to the Os60sP0p in a head/tail to head manner. Our findings provide insights into the functions of OsSec18 in plant growth, vesicle fusion and vacuolar morphology.

In our study, we found three isoforms of OsSec18 in different tissues, clearly suggesting that each isoform may have a unique function in each tissue. The isoform with the highest molecular mass was expressed in leaves, whereas the vacuole morphology and function are largely different from those of other tissues. In addition, the middle size isoform was found in stems and mature seeds, where the vacuoles are transformed into storage- or transportation-related organelles. The smallest isoform was found in inflorescences and immature seeds, which have highly active sites of protein synthesis and cell division. However, the mechanisms by which these isoforms are formed remain unknown. Several mechanisms could underlie the formation of these isoforms. One possible mechanism is alternative splicing at the transcriptional level; another possible mechanism is post-translational modification such as phosphorylation. Thus, our findings introduce new avenues of investigation into the functions of vacuolar fusion in higher plants. It will be interesting to explore the functions of different isoforms of OsSec18 in rice in future.

## Conclusions

In the present study, we found that OsSec18 is a component of a 290-kDa complex in rice endosperm cells, and moreover, Os60sP0 was identified as a component of this complex, revealing that the OsSec18 complex contains another complex of P0(P1-P2)_2_ in rice endosperm cells. Furthermore, we determined that the N-terminus of OsSec18 interacts with the N- and C-termini of Os60sP0, whereas the C-terminus of OsSec18 interacts only with the C-terminus of Os60sP0. We propose a molecular model for the interaction between OsSec18 and Os60sP0.

## Methods

### Materials

The *S. cerevisiae* sec18 mutant sey5186, carrying the genotype *MAT sec18-1 ura3-52 leu2-3, 112 GAL*^*+*^, and wild-type sey6210, carrying the genotype *MAT ura3-52 leu2-3,112 his3-200 trp1-901 lys2-801 suc2-9*, were kindly provided by Karl Fu. The *OsSec18* cDNA clone was purchased from Japanese NIAS GenBank (Accession No. AK072976). A *japonica* variety, TP309, was used in all plant experiments. All biological reagents, including enzymes, kits, and biomaterials, are listed in Additional file 3: Table S1.

### Genetic complementation assays in yeast

A full-length cDNA of rice *Sec18* gene was digested by restriction enzymes, *SacI* and *BamHI*, and then inserted into the *pYES.2* vector (Invitrogen, Carlsbad, CA, USA) and designated as *pOsPMP77*. The plasmid was introduced into the sec18p mutant strain Sey5186 and the wild-type strain Sey6210, following standard protocols [26]. The transformant strains were grown at 37°C for 30 hrs. The sample preparation for electron microscopy was performed as described by Yang *et al.* [27]. The colony phenotypes and cellular microstructures were observed via transmission electron microscopy (FEI Company, Hillsboro, OR, USA).

### Plasmid construction and transgenic plant generation

An overexpression vector driven by the *CaMV35S* promoter was constructed. Briefly, the full-length cDNA encoding OsSec18 (GenBank accession No. J023146P19) was digested using the restriction enzymes *BamHI* and *EcoRI* and then inserted into the PKANNIBLE plasmid vector. The resulting plasmid was designated as *pOsPMP124*. Transgenic plants containing the overexpression plasmids were generated via biolistic bombardment-mediated transformation.

### Antiserum preparations

A serum against OsSec18 was prepared by Shanghai ImmunoGen Biological Technology. The sera against Os60sP0p and Os60sP1p were prepared by Nanjing Genscript Company. Briefly, the full-length *OsSec18* cDNA was inserted into the *pET32a* plasmid for fusion with a His tag. The engineered *E. coli* strain *BL21* was incubated at 30°C for 6 hrs after induction using IPTG. After harvesting the cells, crude protein was extracted in phosphate buffered saline (PBS). After clarification via centrifugation at 6000 g at 4°C, the crude protein was purified using a His-tagged affinity column. The full-length OsSec18 was used as the antigen to inoculate rabbits; these antibodies were generated by Shanghai ImmunoGen Biological Technology (Shanghai, China). For the preparation of antibodies against Os60sP0p and Os60sP1p, the appropriate peptides derived from Os60sP0p and Os60sP1p were synthesized and used as antigens for inoculation of rabbits; these antibodies were prepared by Genscript Company (Nanjing, China).

### SEC and MADLI-TOF mass spectrometry analyses

Rice immature endosperm was harvested after 10–14 days of pollination. Total soluble protein was extracted using PBS containing 0.1% MG132 and 1% protease inhibitor cocktail (Sigma Aldrich, St. Louis, MO, USA). The crude protein extracts were clarified via centrifugation at 10,000 g at 4°C for 10 min. The total protein was filtered through a 0.8-μm filter (Millipore, Billerica, MA, USA). The protein solution was loaded on a Sepharose 300 column (GE Healthcare, Fairfield, CT, USA) and collected in fractions of 2 mL/tube. The protein fractions were separated via 10% SDS-PAGE and then analyzed via Western blot using the appropriate antisera described above. The corresponding proteins and the appropriate molecular mass markers were separated via SDS-PAGE. The proteins of interest were carefully excised following SDS-PAGE and were washed with buffer I (50% v/v acetonitrile, 100 mM ammonium bicarbonate, pH 8.0) and incubated in buffer II (10 mM DTT in 50 mM ammonium bicarbonate, pH 8.0) at 65°C for 1 h, followed by incubation in buffer III (55 mM iodine ammonium acetate, 50 mM ammonium bicarbonate, pH 8.0) in a dark room for 30 min. Enzymatic hydrolysis was performed using trypsin (Promega, Madison, WI, USA) at 37°C overnight after washing with 10 mM ammonium bicarbonate and acetonitrile. An equal volume of buffer IV (60% v/v acetonitrile, 5% formic acid) was added, and then, the sample was ultrasonicated for 10 min. The supernatant was vacuumed dry, and 3 μL of buffer IV was added to dissolve the protein pellets. The resulting protein fractions were used for MADLI-TOF mass spectrometry analysis. The mass spectra were recorded using an Ettan MALDI-TOF/Pro mass spectrometer (ABI, Carlsbad, CA, USA), and the MS data were analyzed using Scaffold software.

### Yeast two-hybrid analysis

A yeast two-hybrid kit was used. Briefly, the full-length and various deletion constructs of the *OsSec18* and *Os60sP0* genes were inserted into *pGBKT7* and *pGADT7* (Clontech, Mountain View, CA, USA) according to the manufacturer’s instructions, and the constructed plasmids were transformed into the yeast strain *AH109* using the LiAc method [28]. The yeast strains were grown in YPDA media (20 g/L tryptophan, 10 g/L yeast extract, 0.03 g/L adenine, 50 mL 40% glucose, 20 g/L agar, pH 5.8) or SD media (Shanghai Genomics, Shanghai, China) at 30°C or 25°C. The transformants were grown on SD medium lacking leucine and tryptophan, on SD medium lacking leucine, tryptophan and histidine or on SD medium lacking leucine, tryptophan, histidine and adenine. A yeast strain co-transformed with *pGBKT7-p53* and *pGADT7-T* was used as a positive control, and a yeast strain co-transformed with *pGBKT7-lam* and *pGADT7-T* was used as a negative control.

### Co-IP and Western blot analysis

The crude protein extracts from immature endosperm (approximately 200 mg) was obtained using 2 mL RIPA buffer (PBS containing 0.1% MG132 and 1% protease inhibitor cocktail, Sigma Aldrich, St. Louis, MO, USA) and then centrifuged at 12,000 g for 10 min at 4°C. The crude protein extracts were pre-precipitated using 80 μL of Protein A agarose beads (Beyotime, Shanghai, China) at 4°C for 1 h. The supernatant was collected via brief centrifugation, and antiserum against OsSec18 or Os60sP0p was added to the supernatant and then rotated at 4°C for 2 h. Then, 60 μL of Protein A agarose beads was added to the supernatant, and the samples were rotated again at 4°C for 1 h. The precipitated protein complexes were separated via centrifugation at 12,000 g at 4°C for 2 min and then washed three times with 1 mL of PBS containing 1 mM PMSF. Then, 20 μL of total protein extraction buffer (66 mM Tris–HCl, pH 6.8, 2% SDS, 2% β-ME) was added to dissolve the pellets. The sample was separated via 12% SDS-PAGE, followed by Western blot analysis using antiserum against OsSec18, Os60sP0p or Os60sP1p. RIPA buffer was used as a negative control for this experiment.

### Electron microscopy

The sample preparation and observation for electron microscopy were performed as described by Yang *et al*. [27].

## Additional files

Additional file 1: Figure S1. Guide Tree of the Sec18 or Pftf gene in tobacco, rice, human and yeast. Additional file 2: Figure S2. Alignment of the amino acid sequences of the Sec18 gene in Pftf, tobacco, rice, human and yeast. Additional file 3.
**List of biological reagents.**
