# Supplementary material for: The OsSec18 complex interacts with P0(P1-P2)2 to regulate vacuolar morphology in rice endosperm cell
Source: BMC Plant Biol. 2015 Feb 17;15:55. doi: 10.1186/s12870-014-0324-1 (PMC4340293; doi:10.1186/s12870-014-0324-1)
Supplement: Additional file 1: Figure S1. — Guide Tree of the Sec18 or Pftf gene in tobacco, rice, human and yeast. [file 12870_2014_324_MOESM1_ESM.doc]

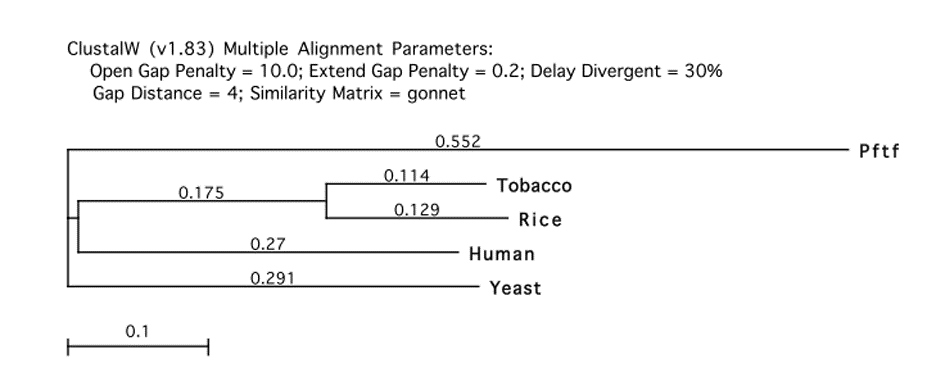


**Fig. S1** **Phylogenetic Tree of the Sec18 gene in red peppers, tobacco, rice, human and yeast. Use Phylogenetic Analyses function of MacVector11.0.4 to analysis the phylogenetic tree of Sec18 genes in five different species.**
