# Supplementary material for: The OsSec18 complex interacts with P0(P1-P2)2 to regulate vacuolar morphology in rice endosperm cell
Source: BMC Plant Biol. 2015 Feb 17;15:55. doi: 10.1186/s12870-014-0324-1 (PMC4340293; doi:10.1186/s12870-014-0324-1)
Supplement: Additional file 2: Figure S2. — Alignment of the amino acid sequences of the Sec18 gene in Pftf, tobacco, rice, human and yeast. [file 12870_2014_324_MOESM2_ESM.doc]

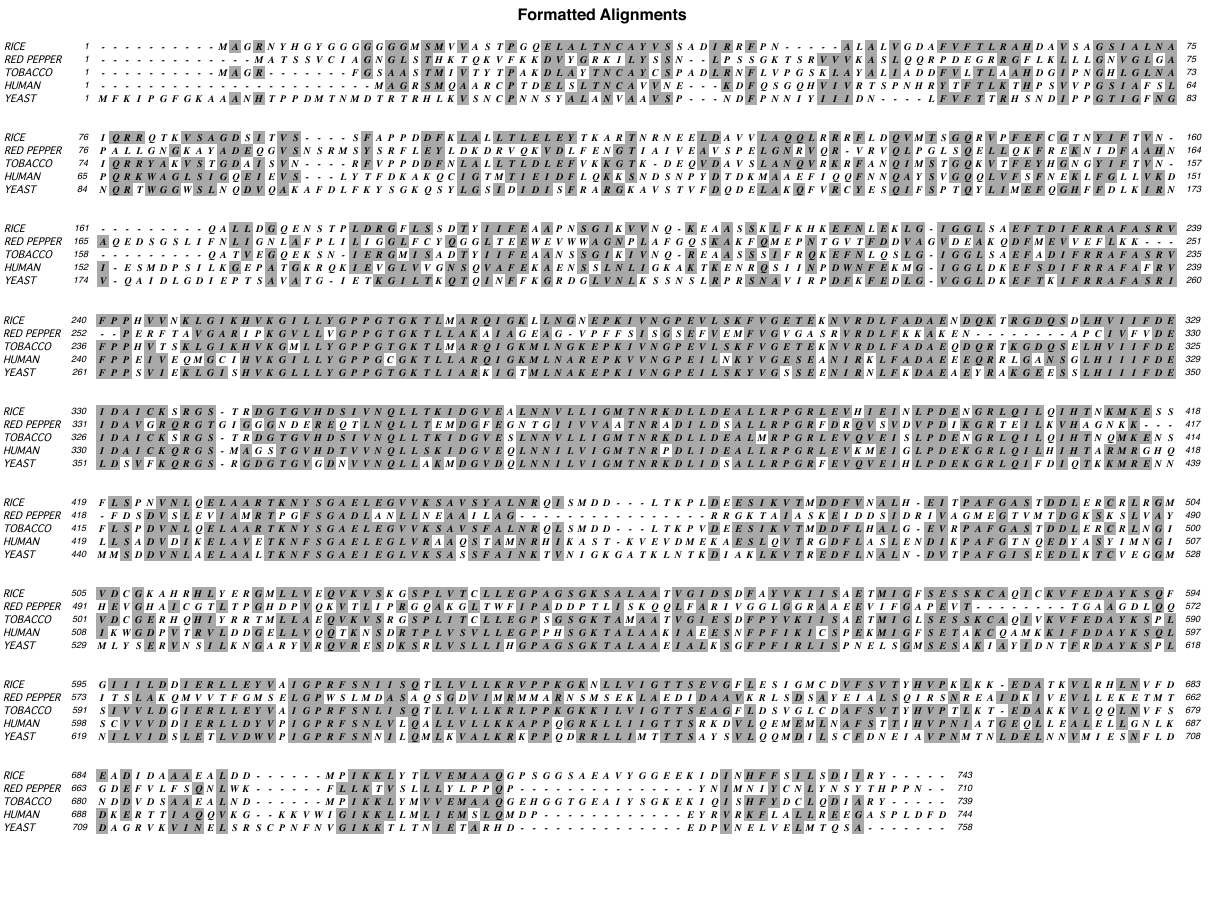


**Fig.S2** **Alignment of the amino acid sequences of the Sec18 gene in red peppers, tobacco, rice, human and yeast.** **Use ClustalW Alignment function of MacVector11.0.4 to analysis the phylogenetic tree of Sec18 genes in five different species.**
