# Supplementary material for: The OsSec18 complex interacts with P0(P1-P2)2 to regulate vacuolar morphology in rice endosperm cell
Source: BMC Plant Biol. 2015 Feb 17;15:55. doi: 10.1186/s12870-014-0324-1 (PMC4340293; doi:10.1186/s12870-014-0324-1)
Supplement: Additional file 3. — List of biological reagents. [file 12870_2014_324_MOESM3_ESM.doc]

| TP309 japonica variety | use for transgene |  |
| --- | --- | --- |
| sey5186 sec18 mutant line | use for yeast complement | |
| sey6210 sec18 wild-type line | use for yeast complement | |
| BL21 E. coli strain | use for antibody preparation | |
| AH109 yeast expression strain | use for yeast two-hybrid analysis | |
| OsSec18 cDNA clone | use for plasmids construct | |
| pYES.2 vector | use for yeast complement plasmids construct | |
| PKANNIBLE plasmid vector | use for transgene plasmids construct | |
| pET32a | use for antibody preparation plasmids construct | |
| pGBKT7 | use for yeast two-hybrid analysis plasmids construct | |
| pGADT7 | use for yeast two-hybrid analysis plasmids construct | |
| pGBKT7-p53 | use for yeast two-hybrid analysis | |
| pGADT7-T | use for yeast two-hybrid analysis | |
| pGBKT7-lam | use for yeast two-hybrid analysis | |
| SacI | use for yeast complement plasmids construct | |
| BamHI | use for yeast complement plasmids construct and transgene plasmids construct | |
| EcoRI | use for transgene plasmids construct | |

**Table S1 List of biological reagents**
